# Effects of Constitutive β-Catenin Activation on Vertebral Bone Growth and Remodeling at Different Postnatal Stages in Mice

**DOI:** 10.1371/journal.pone.0074093

**Published:** 2013-09-16

**Authors:** Min Jia, Sixu Chen, Bo Zhang, Huaping Liang, Jianquan Feng, Zhaowen Zong

**Affiliations:** 1 Department of Trauma Surgery, State Key Laboratory of Trauma, Burn and Combined injury, Daping Hospital, Third Military Medical University, ChongQing, China; 2 Department of Biomedical Sciences, Baylor College of Dentistry, Texas Agriculture and Mechanic Health Science Center, Dallas, Texas, United States of America; Faculté de médecine de Nantes, France

## Abstract

**Background and Objective:**

The Wnt/β-catenin signaling pathway is essential for controlling bone mass; however, little is known about the variable effects of the constitutive activation of β-catenin (CA-β-catenin) on bone growth and remodeling at different postnatal stages. The goal of the present study was to observe the effects of CA-β-catenin on vertebral bone growth and remodeling in mice at different postnatal stages. In particular, special attention was paid to whether CA-β-catenin has detrimental effects on these processes.

**Methods:**

Catnblox(ex 3) mice were crossed with mice expressing the TM-inducible Cre fusion protein, which could be activated at designated time points via injection of tamoxifen. β-catenin was stabilized by tamoxifen injection 3 days, and 2, 4, 5, and 7 months after birth, and the effects lasted for one month. Radiographic imaging, micro-computed tomography, immunohistochemistry, and safranin O and tartrate-resistant acid phosphatase staining were employed to observe the effects of CA-β-catenin on vertebral bone growth and remodeling.

**Results:**

CA-β-catenin in both early (3 days after birth) and late stages (2, 4, 5, and 7 months after birth) increased bone formation and decreased bone resorption, which together increased vertebral bone volume. However, when β-catenin was stabilized in the early stage, vertebral linear growth was retarded, and the mice demonstrated shorter statures. In addition, the newly formed bone was mainly immature and located close to the growth plate. In contrast, when β-catenin was stabilized in the late stage, vertebral linear growth was unaffected, and the newly formed bone was mainly mature and evenly distributed throughout the vertebral body.

**Conclusions:**

CA-β-catenin in both early and late stages of growth can increase vertebral bone volume, but β-catenin has differential effects on vertebral growth and remodeling when activated at different postnatal stages.

## Introduction

The evolutionarily conserved Wnt signaling pathway mainly includes the canonical Wnt/β-catenin and non-canonical pathways that can be further subdivided into planar cell polarity and Wnt/Ca^2+^ pathways. Both canonical and non-canonical signaling pathways participate in many physiological and pathological processes [1-4]. Several lines of evidence suggest that Wnt/β-catenin signaling is essential for bone formation and resorption [4-8]. Activating mutations in lipoprotein receptor-related protein 5 (LRP5), a Wnt co-receptor, induce high bone mass phenotype, whereas inactivating mutations cause osteoporosis-pseudoglioma syndrome, characterized by osteoporosis and blindness [9,10]. Studies in animal models with genetically modified LRP5 protein support these findings [11]. Overexpression of the Wnt ligand inhibitor Dickkopf-1, induced low bone mass and decreased bone formation [12]. In contrast, sclerostin (Wnt ligand inhibitor) knockout mice exhibited increased bone formation and high bone mass [13]. These results suggest that the canonical Wnt signaling pathway plays an important role in controlling bone formation and resorption, thus presenting a potential target for the development of novel bone-building drugs.

Wnt/β-catenin signaling involves multiple steps that may be considered for pharmacological intervention [5,14]. Enhanced Wnt/β-catenin signaling or deficiency/neutralization of Wnt antagonists is associated with increased bone formation and/or decreased bone resorption, suggesting potential therapeutic application in low bone volume conditions [15-22]. However, controversies exist with regard to several issues, including safety, dosage, duration of treatment, and mechanisms of intervention on osteoblastogenesis and osteoclastogenesis [22-26]. To date, there have been no data demonstrating the effects of Wnt/β-catenin signaling pathway on bone growth and remodeling at different postnatal stages. In particular, no data are available on whether manipulation of this signaling pathway might have detrimental effects on bone growth.

The large number of Wnt proteins, receptors, coreceptors, and soluble inhibitors of Wnt signaling makes it difficult to define the specific functions of Wnt /β-catenin signaling. Fortunately, a molecular node of canonical Wnt signaling involves β-catenin with only one encoding gene, which offers the possibility of genetic manipulation. Constitutive activation of β-catenin (CA-β-catenin) can thus be induced in mice expressing a β-catenin mutant allele in which all the serine and threonine residues of exon 3 are phosphorylated by GSK-3β [23]. In the present study, we investigated the effects of CA-β-catenin on vertebral bone growth and remodeling in mice at different stages using pro-collagen I Cre-ER^TM^ as the promoter, which could be activated at designated time-points via injection of tamoxifen (TM) [27,28].

## Materials and Methods

The experimental protocol was reviewed and approved by the Ethical Committee of the Daping Hospital, Third Military Medical University (China).

### Generation of Mice and the Tamoxifen Injection Procedure

Mice expressing the TM-inducible Cre fusion protein, Cre-ER^TM^, under the control of a 3.2 kb mouse pro-collagen 1 promoter (3.2 kb Col1-Cre ER^TM^) active in osteoblasts, odontoblasts and tendon fibroblasts [27,28] were generated by TM injection and crossed with Catnb+/lox(exon 3) mice [23]. Genotyping was performed with a routine method. Briefly, DNA was extracted from the toe of each mouse using a standard protocol, and subjected to PCR for genotyping. The PCR primers for Cre were 5'-CCCGCAGAACCTGAAGATG-3' (sense) and 5'-GACCCGGCAAAACAGGTAG-3' (anti-sense), and the PCR primers for Catnb+/lox(exon 3) mice were 5'-AGGGTACCTGAAGCTCAGCG-3' (sense) and 5'-CAGTGGCTGACAGCAGCTTT-3' (anti-sense).

TM (Sigma-Aldrich, St. Louis, MO, USA) was dissolved in a small volume of ethanol and diluted with corn oil at a concentration of 10 mg/ml [27,28]. CA-β-catenin and wild-type mice (n=6 at each time point) were intraperitoneally injected with TM (75 mg/kg) twice a week for one month. The start times for TM injection were day 3, and months 2, 4, 5, and 7.

Site-specificity of Col1-Cre ER^TM^ was examined by crossing Col1-Cre ER^TM^ mice with ROSA26 reporter mice. Wild-type mice were also crossed with ROSA26 mice to serve as controls. TM was injected as aforementioned. The specificity of Col1-CreER^TM^ expression was confirmed by β-gal staining.

### Radiographic imaging and tissue preparation

At the designated time-points (1, 3, 5, 6 and 8 months after birth), mice were deeply anesthetized and radiographic images of entire skeletons obtained using a small animal X-ray machine (Model 8050-020, Field Emission Corporation, Inc., McMinnville, OR, USA) [29]. After whole-body X-ray was taken, 5 ml of whole blood was removed from the mice by heart puncture. The blood was incubated at room temperature for 30 min, and centrifuged for 5 min at 5000 rpm. The serum was then collected and enzyme-linked immunoassay (ELISA) or radioimmunoassay (RIA) was performed to determine the concentration of intact N-terminal propeptide of type I procollagen (P1NP), tartrate resistant acid phosphatase 5b (TRAP5b), osteocalcin, C-terminal telopeptides of type I collagen (CTX-I), according to the manufacturer’s instructions (Immunodiagnostic Systems Ltd, Boldon, UK).

After whole-body x-ray was performed and whole blood was taken, the mice were sacrificed by overdose of anesthesia and the spines were removed and fixed in 4% paraformaldehyde at 4°C overnight. The fifth lumbar vertebra was cut and saved for micro-computed tomography (microCT) examination and real-time PCR. The samples that were left were decalcified in 10% ethylenediaminetetraacetic acid at 4°C for 7 days. X-ray imaging was used to determine if the samples were adequately decalcified. Then, the third and fourth lumbar vertebrae were tissue-processed, embedded in paraffin, and sectioned at a thickness of 5 µm horizontally or coronally. The sections were de-paraffined and rehydrated, and were used for hematoxylin and eosin (H&E) staining, immunohistochemistry, TRAP staining, and safranin O staining.

### Real-time PCR

Total RNA was extracted from 100 mg of the vertebral trabecular bone using Tripure^TM^ (Promega Corp, Madison, WI) according to the manufacturer’s instructions. The amount of RNA was quantified by spectrophotometry, after which cDNA was prepared from 1.0 µg RNA. The RNA was digested with DNase to eliminate any contaminating genomic DNA before quantitative real-time RT-PCR was performed. Then SYBR green detection method was used to examine the expression of receptor activator of nuclear factor kappa B ligand (RANKL), osteoprotegerin (OPG), TRCAP, tissue non-specific alkaline phosphatase (TNAP), RunX2, and bone sialoprotein (BSP). Glyceraldehyde-3-phosphatedehydrogenase (GAPDH) served as a control and the expression of a given gene was expressed as proportion relative to the average value of GAPDH. The relative ratio of RANKL:OPG expression was calculated and used for statistics. The primers used were sythesized by Sangon Biotech (Shang Hai, China) and are shown in Table 1.

**Table 1:** Primers used for real-time PCR.

**Genes**	**Primers**
BSP	F: 5’-CAATCCGTGCCACTCACT-3’, R: 5’-CAAACTCCAAGCCAAAGC-3’
GAPDH	F: 5’-TCACTGCCACCCAGAAGA -3’, R: 5’-AAGTCGCAGGAGACAACC -3’
OPG	F: 5’- GCATTATGACCCAGAAACT -3’, R: 5’- ACCTGAGAAGAACCCATC-3’
RANKL	F: 5’- AACCAAGATGGCTTCTATTACC-3’, R: 5’- AAGGGTTGGACACCTGAATG-3’
RunX2	F: 5’-AGTCCCAACTTCCTGTGCT-3’, R: 5’-GGTGAAACTCTTGCCTCGTC-3’
TNAP	F: 5’- ACGAGATGCCACCAGAGG-3’, R: 5’- AGTTCAGTGCGGTTCCAG -3’
TRAP	F: 5’- GCCCTTACTACCGTTTGC-3’, R: 5’-TCTCGTCCTGAAGATACTGC-3’

### MicroCT examination

The fifth lumbar vertebrae and tibia of mice from each group were dissected and scanned in a micro-CT imaging system (Scanco Medical, Bassersdorf, Switzerland), as described previously [29]. The scanning medium was ethanol, the x-ray tube potential was 45 kVp, and the voxel size was 10 µm^3^. Images were reconstructed and analyzed with EVS Beam software using a global threshold of 1400 Hounsfield units. Total vertebral bone volume fraction was calculated as the percentage of bone volume to total volume (BV/TV) of fifth lumbar vertebra. The mean trabecular thickness (Tb.Th), trabecular separation (Tb. Sp), and trabecular number (Tb.N) were also calculated [30]. As for the tibia, quantitative morphometry data were based on region of interest as following: trabecular bone region starting from growth plate reference level extending 44 slices (0.8 mm) distally, while cortical bone starting from mid-diaphysis extending 22 slices (0.4 mm) proximally. BV/TV, Tb.Th, Tb. Sp and Tb.N were qualified in trabecular bone region of tibia, while average cortical thickness (Ct.Th), total cross-sectional area (Tt.Ar), cortical bone area (Ct.Ar), cortical area fraction (Ct.Ar/Tt.Ar) were qualified in cortical bone region of tibia.

### Immunohistochemical analysis

Immunohistochemistry was performed according to a previous report [31]. Briefly, 5-µm sections were blocked with 10% H_2_O_2_ at room temperature for 10 min, followed by treatment with 3% bovine serum albumin (BSA) at room temperature for 1 h. Sections were incubated with primary antibodies diluted at the appropriate concentrations in 2% BSA/0.1 M phosphate buffered saline (PBS) overnight at 4°C, washed in PBS, and further incubated with a biotinylated IgG antibody at room temperature for 1 h. Next, slides were treated with ABC reagents (Boster, Wuhan, China), and signals for antibody binding visualized with diaminobenzidine (Boster) substrate. Counterstaining was performed with Methyl Green. Slides were fully rinsed with 0.1 M PBS or distilled water between individual steps.

The primary antibodies used included goat TNAP (1:200), goat polyclonal Runx2 (1:300) and rabbit anti- BSP (1:300). All primary antibodies were purchased from Santa Cruz Biotechnology, Inc (CA, USA), and biotinylated goat anti-mouse, rabbit anti-goat, and goat anti-rabbit IgG acquired from Boster. To obtain the percentage of cells expressing a given marker protein, pictures of each section were taken by an Olympus microscope and digital camera under a 200x magnification. The number of specific antigen-positive cells was counted in five random fields. The average percentage of positive cells and standard deviation were calculated in each group and used for statistics.

### Safranin O Staining Protocol

Rehydrated sections were stained with Weigert’s iron hematoxylin for 1 min and rinsed in distilled water until clear. Sections were stained sequentially with 0.02% Fast Green for 5 min, 1% acetic acid for 30 s, and 0.1% Safranin O for 20 min. Slides were not rinsed between steps. Subsequently, slides were rinsed in 95% alcohol for 2 min, 100% alcohol for 3 min (twice), xylene for 2 min (twice), and coverslipped with Permount. All chemicals were purchased from Chuandong Corporation (Chongqing, China).

### TRAP staining

Two coplin jars (A and B) were pre-heated to 37°C with 50 ml stock basic incubation medium (sodium acetate anhydrous, 9.2 g; sodium tartrate dibasic dehydrate, 11.4 g; and glacial acetic acid, 2.8 ml dissolved in 1000 ml of distilled water and the pH was adjusted to 4.7-5.0 with 5M sodium Hydroxide). Then, 50 µl of 2% naphthol AS-BI phosphate substrate in ethylene glycol monoethyl ether was added to jar A, into which the slides were added, and incubated at 37°C for 45 min. A few minutes before the 45 min elapsed, 1 ml of 5% pararosaniline chloride and 1 ml of 4% sodium nitrite was mixed for 30 sec, incubated at room temperature for 2 min without mixing, and then transferred into jar B and mixed well. The slides from jar A were then moved into jar B without rinsing. Incubation at room temperature was done for 1-3 min until the color developed, followed by rinsing and counterstaining with methyl green for 5 min, dehydration, and covering with Permount. All chemicals were purchased from the Chuandong Corporation (Chongqing, China).

Pictures of each section were taken under a magnification of 200×, and the number of TRAP-positive cells was counted in five random fields. The average number and standard deviation of TRAP-positive cells were calculated and used for statistical analyses

### Statistical analysis

All data were expressed as means ± standard error. Statistical significance was evaluated using ANOVA with SPSS11.0 software. Data were considered significant at *P*<0.05.

## Results

### Effect of CA-β-catenin on vertebral bone growth at different stages after birth

The site specificity of Col1-CreER^TM^ expression was confirmed by β-gal staining using ROSA26 mice. Positive staining was mainly found in osteoblasts, but not in the chondrocytes of the growth plate (Figure S1).

**Figure 1:**
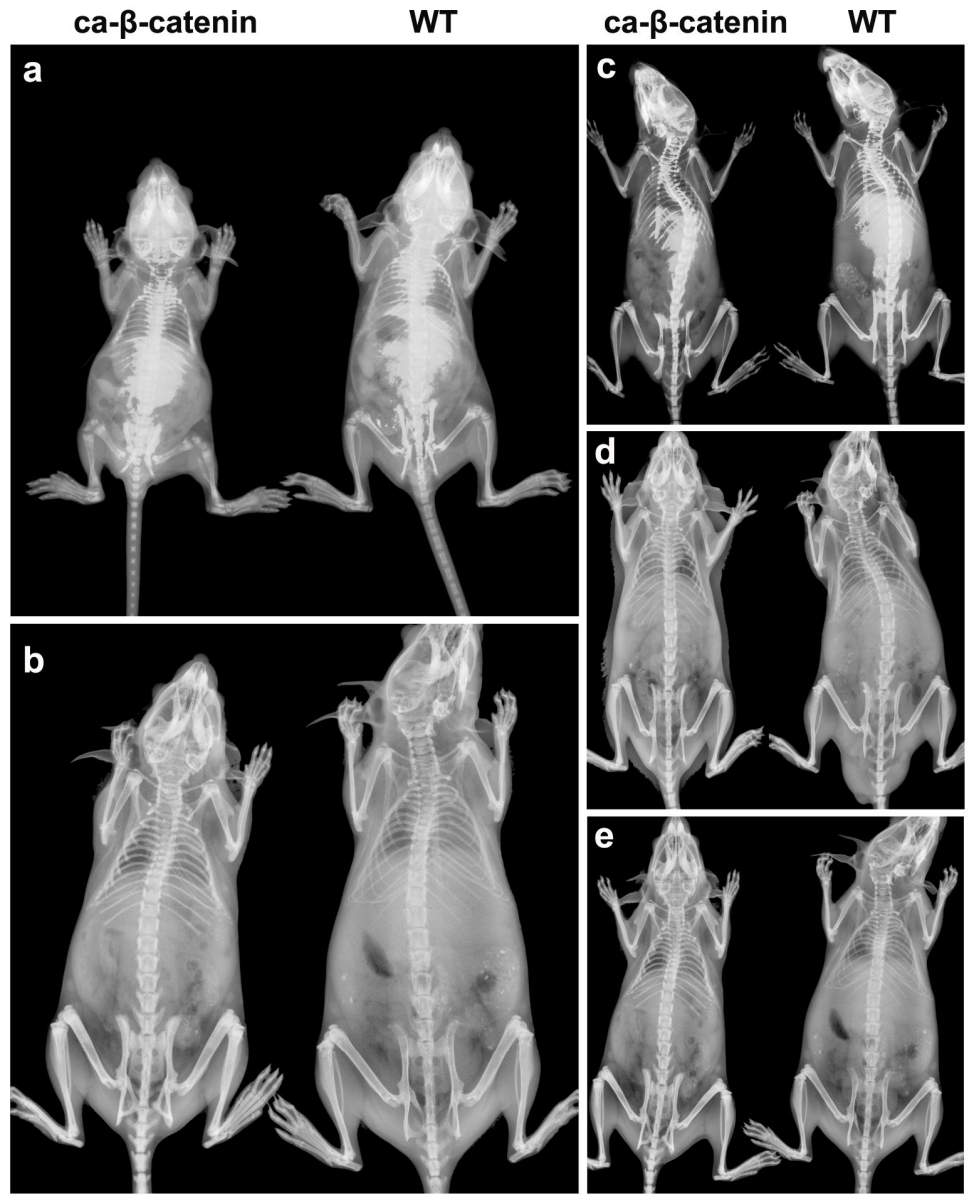
Representative x-ray images of the spine. a-e X-ray images of the spine in wild-type and CA-β-catenin mice 1 month (a), 3 months (b), 5 months (c), 6 months (d) and 8 months (e) after birth.

During the period that we performed our experiments, wild-type mice, Col-Cre mice, and β-catenin exon 3 fx +/+ mice (without Col I Cre) were injected with tamoxifen. The phenotypes of Col-Cre and β-catenin exon 3 fx +/+ mice (without Col I Cre) were the same as wild-type mice (data not shown). We feared that this data would make the manuscript too complicated, so only data from wild-type mice were presented as control in the current study. When β-catenin was stabilized with Col1-CreER^TM^ as a promoter from day 3 of life, mice displayed shorter stature in their first month of life, compared with wild-type mice. In the group with β-catenin stabilization from month 2 to 3, mouse height was still slightly shorter than that of wild-type, but not to a significant extent. Interestingly, when β-catenin was stabilized from months 4, 5 and 7 of life, statures were similar to that in wild-type mice (Figure 1).

**Figure 2:**
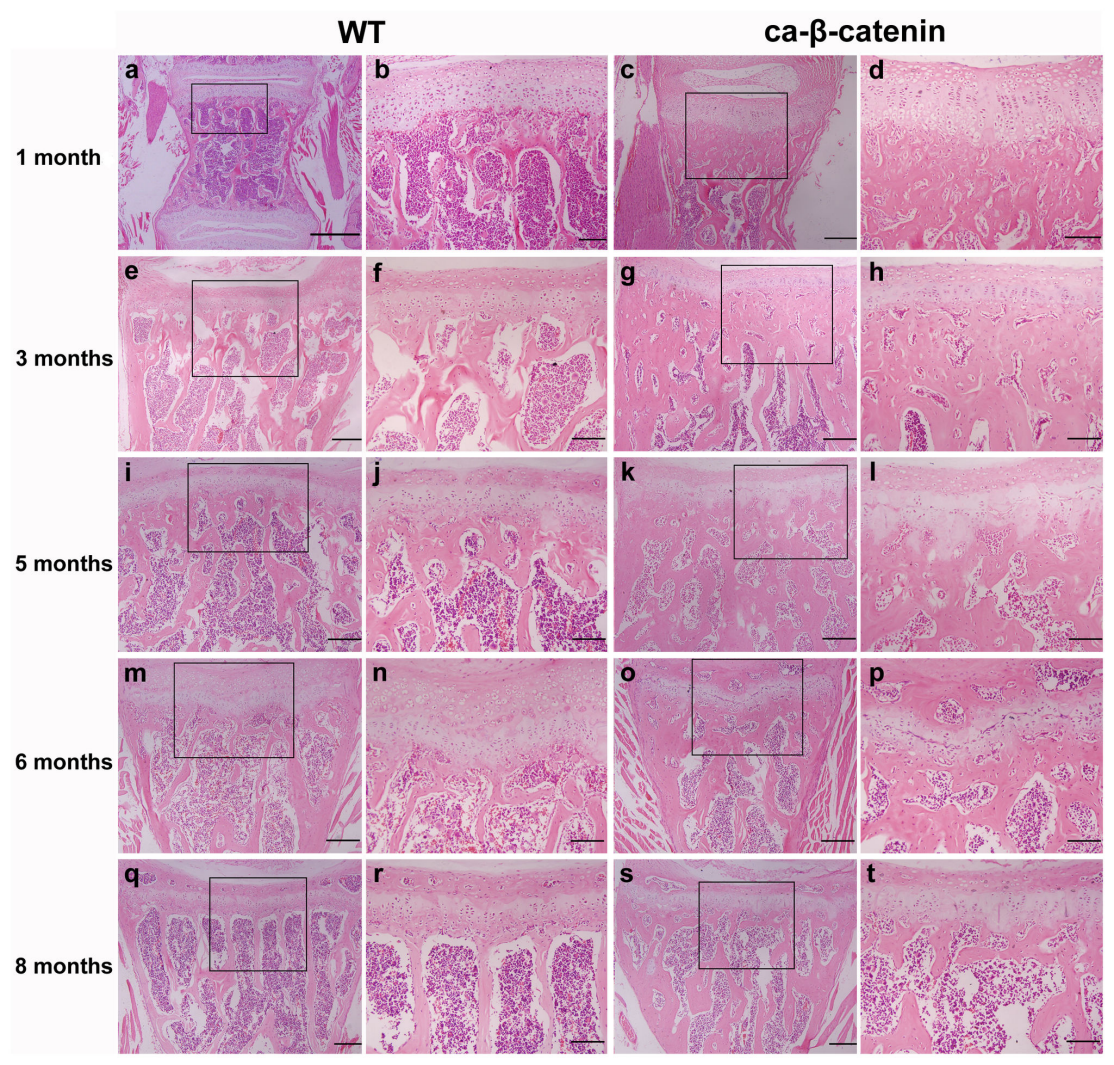
H&E staining of coronal sections of the fourth lumbar vertebra in wild-type and CA-β-catenin mice. Boxes in a, c, e, g, i, k, m, o, q and s are magnified in b, d, f, h, j, l, n, p, r and t, respectively. *Bars*: 200 µm (a, c, e, g, I, k, m, o, q and s) and 100 µm (b, d, f, h, j, l, n, p, r and t).

**Figure 3:**
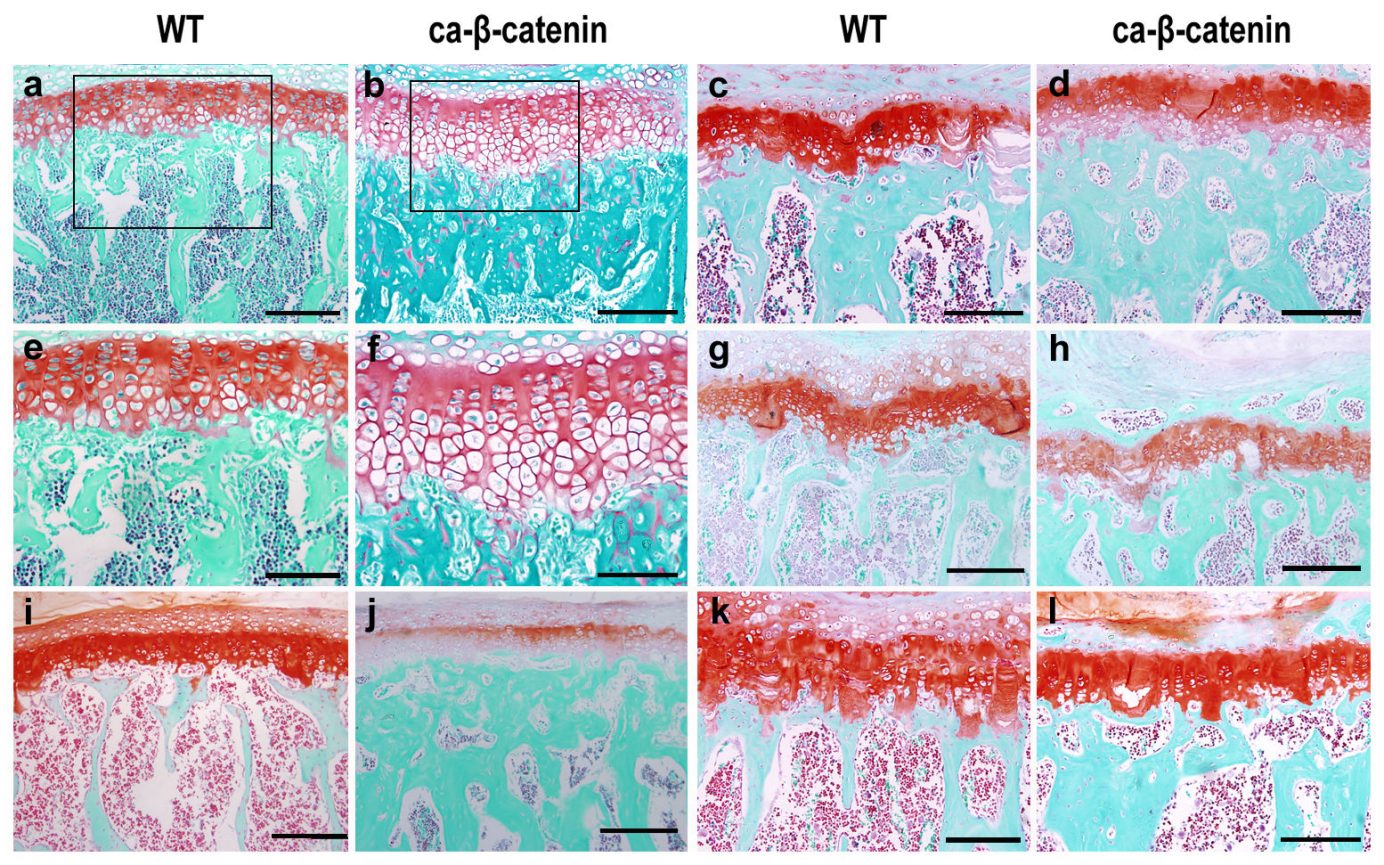
Safranin O staining of lumbar vertebra. a-i Safranin O staining of coronal sections of the fourth lumbar vertebra in wild-type and CA-β-catenin mice at 1 month (a, b, e and f), 3 months (i and j), 5 months (c and d), 6 months (g and h) and 8 months (k and l) of age. Boxes in a and b are magnified in e and f, respectively. Bars: 200 µm (a, b, c, d, g, h, i, j, k and l) and 100 µm (e and f).

Growth plate is the structure responsible for linear growth in vertebrates. H&E and Safranin O staining showed that growth plates in mice with β-catenin stabilization from day 3 were longer than that in wild-type mice (Figures 2 and 3). The hypertrophic layer of the growth plate was the most enlarged part. In contrast, β-catenin stabilization at months 4, 5, and 7 of life had no evident effect on growth plate length (Figures 2 and 3). These results clearly indicate that stabilization of β-catenin at early stages after birth hinders vertebral linear growth in mice.

Similar findings were observed in the long bone. When β-catenin was stabilized from day 3 of life, the lengths of the tibia and femur were shorter than those in wild-type mice, and the hypertrophic layer of the growth plate was enlarged (Figure 1, Figure S2). In contrast, when β-catenin was stabilized 4, 5, and 7 months after birth, the lengths of the long bone and growth plate were not affected (Figure 1, Figure S2).

### Effects of CA-β-Catenin on Vertebral Bone Volume at Different Stages after Birth

**Figure 4:**
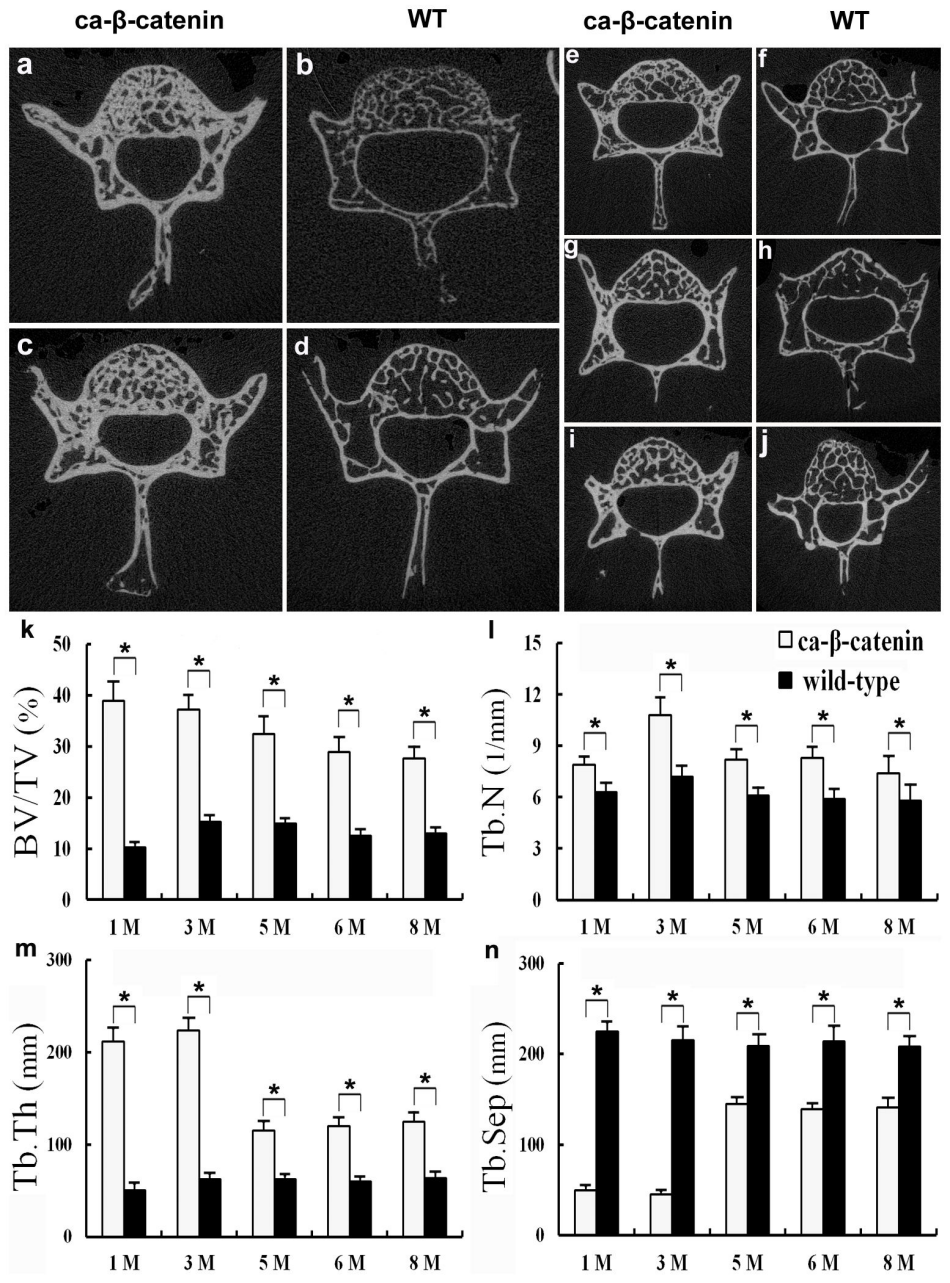
MicroCT examination of lumbar vertebrae in each group. a-j representative, transverse MicroCT images of the fifth lumbar vertebra in wild-type and CA-β-catenin mice at 1 month (a and b), 3 months (c and d), 5 months (e and f), 6 months (g and h) and 8 months (i and j) of age. k MicroCT analysis of BVF (BV/TV, %) of the fifth lumbar vertebra in CA-β-catenin mice and wild-type mice. *BV* trabecular bone volume (mm^3^); *TV* total volume selected for analysis (mm^3^). l Trabecular number (Tb.N) of the fifth lumbar vertebra in CA-β-catenin and wild-type mice. m Mean trabecular thickness (Tb.Th) of the fifth lumbar vertebra in CA-β-catenin and wild-type mice. n Trabecular separation (Tb. Sp) of the fifth lumbar vertebra in CA-β-catenin and wild-type mice. Bars represent the mean ± SEM (n=6 for each group). *p<0.05.

The Wnt/β-catenin signaling pathway plays an important role in controlling bone volume. Our experiments disclosed significantly increased trabecular bone volumes in mice with constitutive activation of β-catenin at both early (day 3) and late stages (months 2, 4, 5 and 7), compared to wild-type mice (*P* <0.05, Figure 4, Figure S3). In addition, Tb.N and Tb.Th significantly increased in the vertebrae of CA-β-catenin mice, and Tb. Sp significantly decreased in the vertebrae of CA-β-catenin mice (*p*<0.05, Figure 4). Except for the Tb.Th, Tb.N and Tb. Sp showed similar change in tibia of CA-β-catenin mice (Figure S3).

However, the location and extent of maturation of excessive formed bone varied with different stages of β-catenin stabilization. Upon induction of β-catenin stabilization from day 3 to month 1 of life, excessive formed bone was located in close proximity to the growth plate, while mice with β-catenin stabilization at the late stages (2, 4, 5 and 7 months after birth) displayed even distribution of excessive bone in the vertebral body (Figure 2).

Safranin O is commonly employed for proteoglycan staining. During rapid bone formation, immature bone stains positive for Safranin O [32,33]. Our data showed that the majority of cancellous bone in vertebral bodies of CA-β-catenin mice at month 1 was positive for Safranin O, indicating that excessive formed bone in vertebrae is immature. In contrast, at later stages (2, 4, 5 and 7 months after birth), excessive formed bone was negative for Safranin O staining (Figure 3).

Similar findings were observed in the long bone (Figure S3). Specifically, CA-β-catenin in both the early (3 days after birth) and late stages (2, 4, 5, and 7 months after birth) increased trabecular bone volume in the long bone (Figure S3). However, when β-catenin was stabilized from day 3 of life, most cancellous bone in the long bone were Safranin-O positive. In contrast, at later stages (2, 4, 5 and 7 months), most of the excessive formed bone were negative for Safranin O staining (Figure S4). CA-β-catenin had no obvious effect on cortical bone (Figure S5).

### Increased bone formation and decreased bone resorption contribute to excessive bone volume in CA-β-catenin mice

**Figure 5:**
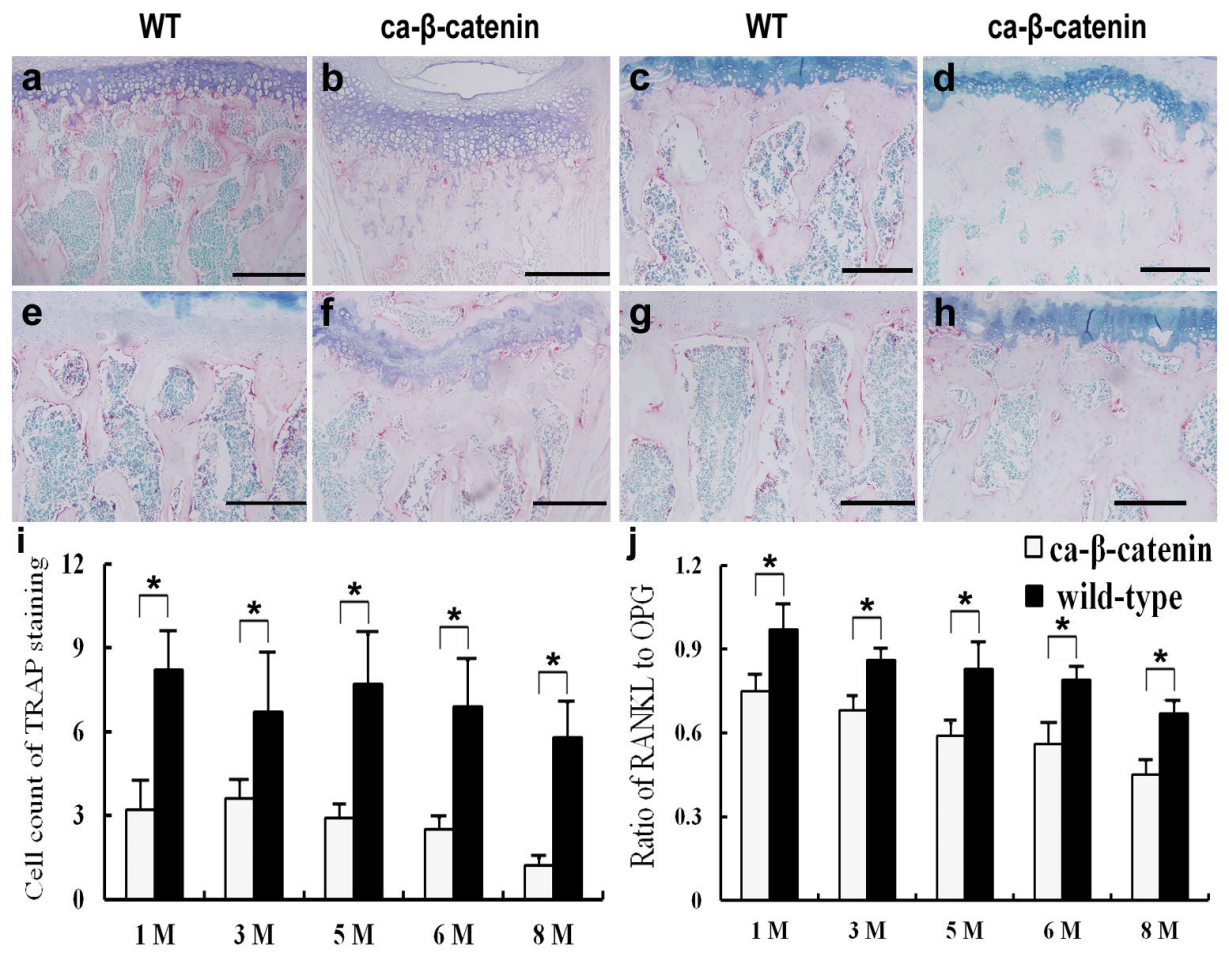
TRAP staining of lumbar vertebra in each group. a-j TRAP staining of coronal sections of the fourth lumbar vertebra in wild-type and CA-β-catenin mice at 1 month (a and b), 3 months (c and d), 6 months (e and f), and 8 months (g and h) of age. Bars: 200 µm. i: The number of TRAP-positive cells in each group. j: The ratio of RANKL:OPG expression levels in each group. Bars represent the mean ± SEM (n=6 for each group). *p<0.05.

**Figure 6:**
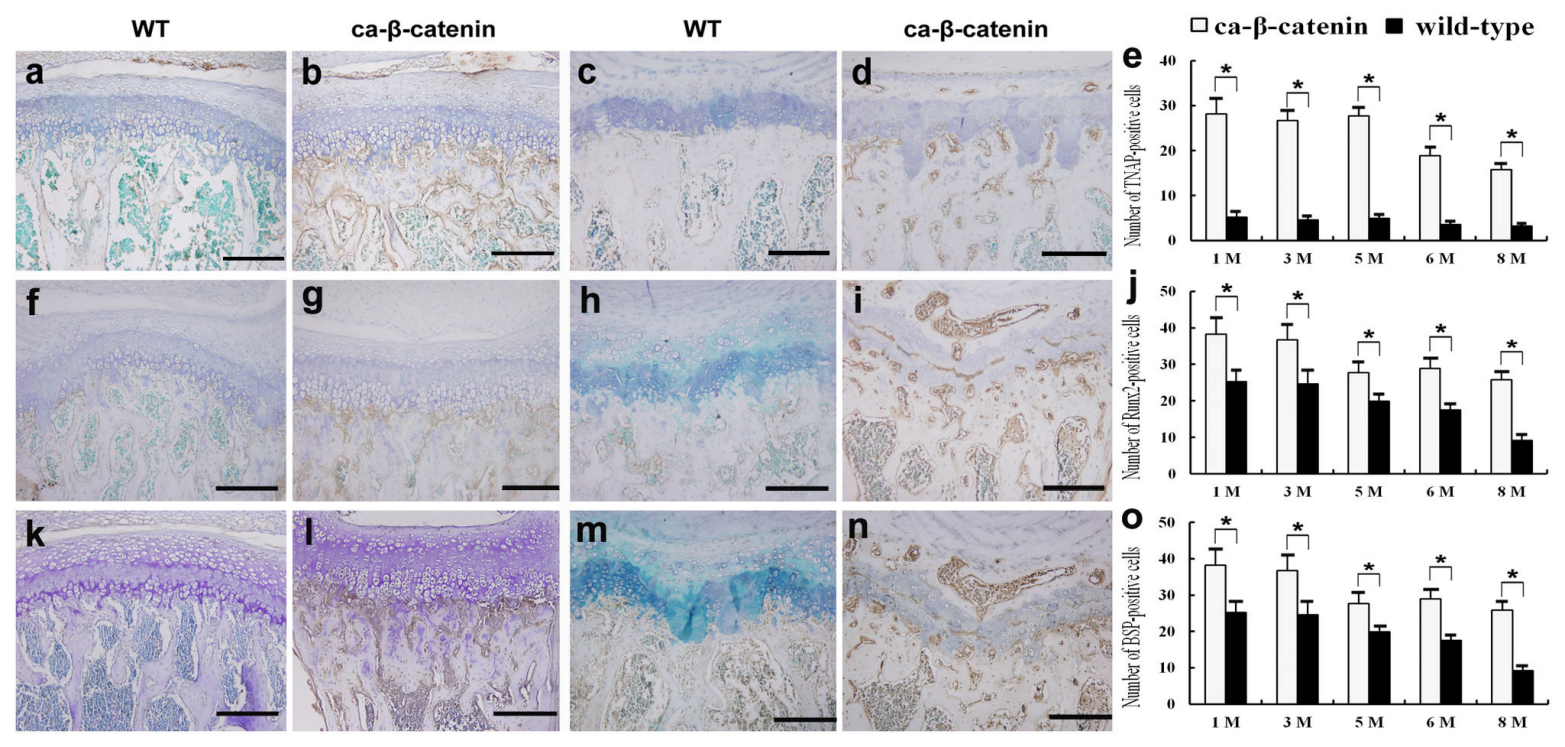
Examination of markers for bone formation and maturation by immunohistochemistry (IHC). a-d IHC against TNAP in wild-type and CA-β-catenin mice at 1 month (a, b) and 6 months (c, d) of age. e: Number of TNAP-positive cells in each group. f-i IHC against RunX2 in wild-type and CA-β-catenin mice at 1 month (f, g) and 6 months (h, i) of age. j: Number of RunX2-positive cells in each group. k-n IHC against BSP in WT and CA-β-catenin mice at 1 month (k, l) and 6 months (m, n) of age. o Number of BSP-positive cells in each group. Bars: 200 µm. Bars represent the mean ± SEM (n=6 for each group). *p<0.05.

The balance of bone formation and bone resorption controls bone volume. TRAP staining showed that the number of osteoclasts in CA-β-catenin mice was lower than that in wild-type mice in both early and late postnatal stages (*p*<0.05, Figure 5A-I, Figure S6). RANKL was an important factor during the maturation of osteoclasts, and OPG was its decoy receptor. Real-time PCR showed that the RANKL:OPG ratio significantly decreased in CA-β-catenin mice at each time point compared with control mice (Figure 5J), which contributed to the decreased number of osteoclasts in CA-β-catenin mice. TNAP served as a useful marker of bone formation. IHC analyses showed higher expression of TNAP protein in ca-β-catenin than wild-type mice, indicative of increased bone formation ability (*p*<0.05, Figure 6A-E). Runx2 and BSP are markers for osteoblasts at the early and late stages, respectively [29,33]. Notably, the numbers of both Runx2 and BSP-positive cells in CA-β-catenin mice were greater than those in wild-type mice in both early and late postnatal stages (*p*<0.05, Figure 6F-J and Figure 6K-O).

**Figure 7:**
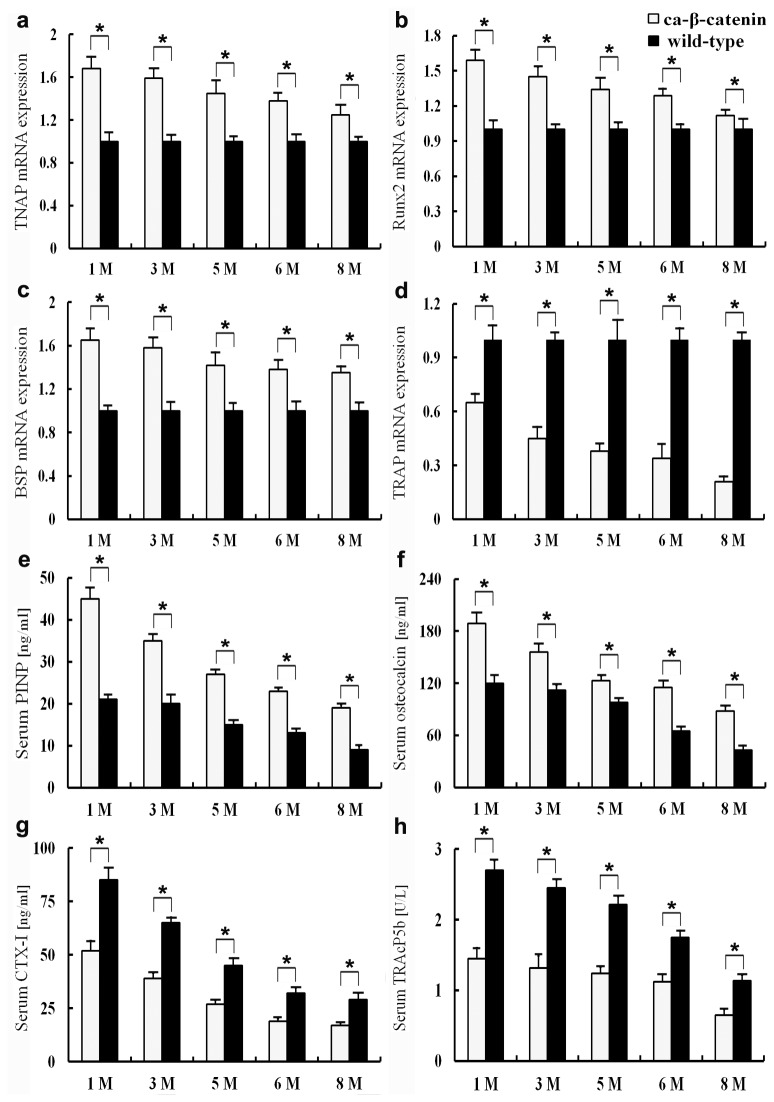
Examination of markers for bone formation and bone resorption by Real-time PCR and EILISA. a The mRNA expression level of tissue non-specific alkaline phosphatase (TNAP) in each group. b The mRNA expression level of Runx2 in each group. c The mRNA expression level of bone sialoprotein (BSP) in each group. d The mRNA expression level of tartrate resistant acid phosphatase (TRAP) in each group. e The serum concentration of intact N-terminal propeptide of type I procollagen (P1NP) in each group. f The serum concentration of osteocalcin in each group. g The serum concentration of C-terminal telopeptides of type I collagen (CTX-I) in each group. h The serum concentration of tartrate resistant acid phosphatase 5b (TRAP5b) in each group. Bars represent the mean ± SEM (n=6 for each group). *p<0.05.

The results of real-time PCR and ELISA further supported the histology findings. As shown in Figure 7A-C, the mRNA expression levels of Runx2, BSP, and TNAP significantly increased in CA-β-catenin mice compared with that in wild-type mice. PINP and osteocalcin are markers of bone formation, while CTX-I and TRAcP5b are markers of bone resorption. It was found that PINP and osteocalcin serum concentrations significantly increased in CA-β-catenin mice, whereas CTX-I and TRAcP5b concentrations significantly decreased (*p*<0.05, Figure 7).

Taken together, these results indicate that stabilization of β-catenin in both early and late postnatal stages increases vertebral bone formation and decreases bone resorption.

## Discussion

Accumulating evidence supports the significance of Wnt/β-catenin signaling in bone formation and bone resorption. However, no data are available about the effect of the Wnt/β-catenin signaling pathway on bone growth and remodeling at different stages after birth. In the current study, we showed that constitutive β-catenin activation at both the early and late stages of growth results in increased bone formation and decreased bone resorption, which concertedly increase vertebral bone volume. However, the stabilization of β-catenin at early and late stages had differential effects on vertebral growth.

The current study found that CA-β-catenin in both the early and late stages of growth increases bone volume by increasing bone formation and decreasing bone resorption. However, controversies exist regarding the role of the Wnt/β-catenin signaling pathway in osteoblastogenesis and osteoclastogenesis [22-26]. Using a mouse model with a Lrp6rs/rs mutation, Kubota et al. showed that mutant mice have low bone mass, owing to accelerated bone resorption [11]. Similar results were observed with CA-β-catenin mice [23]. However, oral administration of AZD2858, a GSK-3 inhibitor, had no influence on the number of osteoclasts in mice [22]. Glass and colleagues suggested this difference might be caused by the fact that Wnt/β-catenin signaling in osteoblasts at different stages has variable effects on osteoclastogenesis [23]. Specifically, the Wnt/β-catenin pathway in mature osteoblasts negatively regulates osteoclastogenesis, but exerts different effects in early osteoblasts and mesenchyaml stem cells. In the current study, a 3.2 kb Col I Cre that mainly activates Wnt/β-catenin in mature osteoblasts was employed [27,28], resulting in suppression of osteoclastogenesis.

Although controversy exists, the effects of Wnt/β-catenin on bone formation are generally considered positive [19,25,34,35]. Wnt6, Wnt10a and Wnt10b inhibit adipogenesis and stimulate osteoblastogenesis through a β-catenin-dependent mechanism [19]. Moreover, oral administration of different types of GSK-3 inhibitors leads to increased bone formation [20,22]. Recently it was shown by Chen and colleagues that deletion of β-catenin in osteoblasts greatly reduced bone formation activity and indirectly increased osteoclast number and activity [36]. Consistent with that finding, the current study found that CA-β-catenin in both the early and late stages increased bone formation, which worked together with decreased bone resorption to increase bone volume.

Another finding of the present study is that stabilization of β-catenin at early and late stages has differential effects on vertebral growth. When β-catenin was activated at the early stages (from day 3 after birth up to 1 month), maturation of vertebral growth plate was retarded in addition to increased bone formation, and mice displayed shortened statures. In contrast, when β-catenin was activated at the late stages (2, 4, 5 and 7 months after birth), growth plates were not significantly affected. Our results indicate that constitutive activation of β-catenin at the early stages has a detrimental effect on vertebral growth. Furthermore, excessive formation of bone in early activation mainly occurs close to the growth plate and involves immature bone (staining positive for Safranin O), while excessive bone formed in the late stages is evenly distributed in the vertebral body and mature (staining negative for Safranin O). Until now, the precise mechanisms underlying the differential effects on vertebral growth by CA-β-catenin at different postnatal stages have remained unknown. Since the promoter used in the current study, pro-collagen I Cre-ER^TM^, is mainly expressed in osteoblasts, but not in chondrocytes, the effect of CA-β-catenin on the growth plate must be indirect. Our preliminary study showed that CA-β-catenin mice demonstrated hypophosphatemia, and a high phosphate diet could partially rescue the phenotype of enlarged growth plate in mice when β-catenin was stabilized at early stage (data not shown). These data indicated that hypophosphatemia might be the reason for retarded linear growth in CA-β-catenin mice. Future studies are needed to investigate the reason for these differential effects. However, regardless of the underlying mechanisms, these discrepancies should be addressed before therapeutics are used that target the Wnt/β-catenin signaling pathway.

In conclusion, the present study shows that CA-β-catenin at different stages after birth can increase vertebral bone volume. This increased bone volume was the result of increased bone formation and decreased bone resorption caused by CA-β-catenin. However, CA-β-catenin induced at different stages has differential effects on vertebral linear growth, as well as distribution and maturation of the newly formed bone.

## Supporting Information

Figure S1 **Site-specificity of Col1-CreER^TM^.** a Col 1 Cre expression was not found in growth plate in wild-type mice crossed with ROSA26 mice. b Col 1 Cre expression was not found in growth plate in Col1-CreER^TM^ mice crossed with ROSA26 mice. c Col 1 Cre expression was not found in osteoblasts in wild-type mice crossed with ROSA26 mice. d Col 1 Cre expression was found in osteoblasts in Col1-CreER^TM^ mice crossed with ROSA26 mice. Bars: 200 µm.
(TIF)

Figure S2 **H&E staining of coronal sections of the tibia in wild-type and CA-β-catenin mice.** Boxes in a, c, e, g, I, k, m, o, q and s are magnified in b, d, f, h, j, l, n, p, r and t, respectively. Bars: 200 µm (a, c, e, g, I, k, m, o, q and s) and 100 µm (b, d, f, h, j, l, n, p, r and t).
(TIF)

Figure S3 **MicroCT examination of trabecular bone of proximal tibia in each group.** a-j representative, transverse MicroCT images of the trabecular bone in CA-β-catenin and wild-type mice at 1 month (a and b), 3 months (c and d), 5 months (e and f), 6 months (g and h) and 8 months (i and j) of age. k MicroCT analysis of BVF (BV/TV, %) of the proximal tibia in CA-β-catenin mice and wild-type mice. *BV* trabecular bone volume (mm^3^); *TV* total volume selected for analysis (mm^3^). l Trabecular number (Tb.N) of the proximal tibia in CA-β-catenin and wild-type mice. m Mean trabecular thickness (Tb.Th) of the proximal tibia in CA-β-catenin and wild-type mice. n Trabecular separation (Tb. Sp) of the proximal tibia in CA-β-catenin and wild-type mice. Bars represent the mean ± SEM (n=6 for each group). * *p*<0.05.
(TIF)

Figure S4 **Safranin O staining of tibia in each group.** a-h Safranin O staining of coronal sections of tibia in wild-type and CA-β-catenin mice at 1 month (a and b) 3 months (c,d g and h) 5 months (e and f), 6 months (i and j) and 8 months (k and l) of age. Boxes in c and d are magnified in g and h, respectively. Bars: 200 µm (a, b, c, d, e, f, i, j, k and l) and 100 µm (g and h).
(TIF)

Figure S5 **MicroCT examination of cortical bone of tibia in each group.** a-j representative, transverse MicroCT images of the cortical bone in CA-β-catenin and wild-type mice at 1 month (a and b), 3 months (c and d), 5 months (e and f), 6 months (g and h) and 8 months (i and j) of age. k Total cross-sectional area (Tt.Ar) in CA-β-catenin and wild-type mice. l Cortical bone area (Ct.Ar) in CA-β-catenin and wild-type mice. m Cortical area fraction (Ct.Ar/Tt.Ar) in CA-β-catenin and wild-type mice. n Average cortical thickness (Ct.Th) in CA-β-catenin and wild-type mice. Bars represent the mean ± SEM (n=6 for each group). *p<0.05.
(TIF)

Figure S6 **TRAP staining of tibia in each group.** a-h TRAP staining of coronal sections of tibia in wild-type and CA-β-catenin mice at 1 month (a and b), 3 months (c and d), 6 months (e and f), and 8 months (g and h) of age.
(TIF)
