# Supplementary figures and images for: Effects of Constitutive β-Catenin Activation on Vertebral Bone Growth and Remodeling at Different Postnatal Stages in Mice
Source: PLoS One. 2013 Sep 16;8(9):e74093. doi: 10.1371/journal.pone.0074093 (PMC3774640; doi:10.1371/journal.pone.0074093)

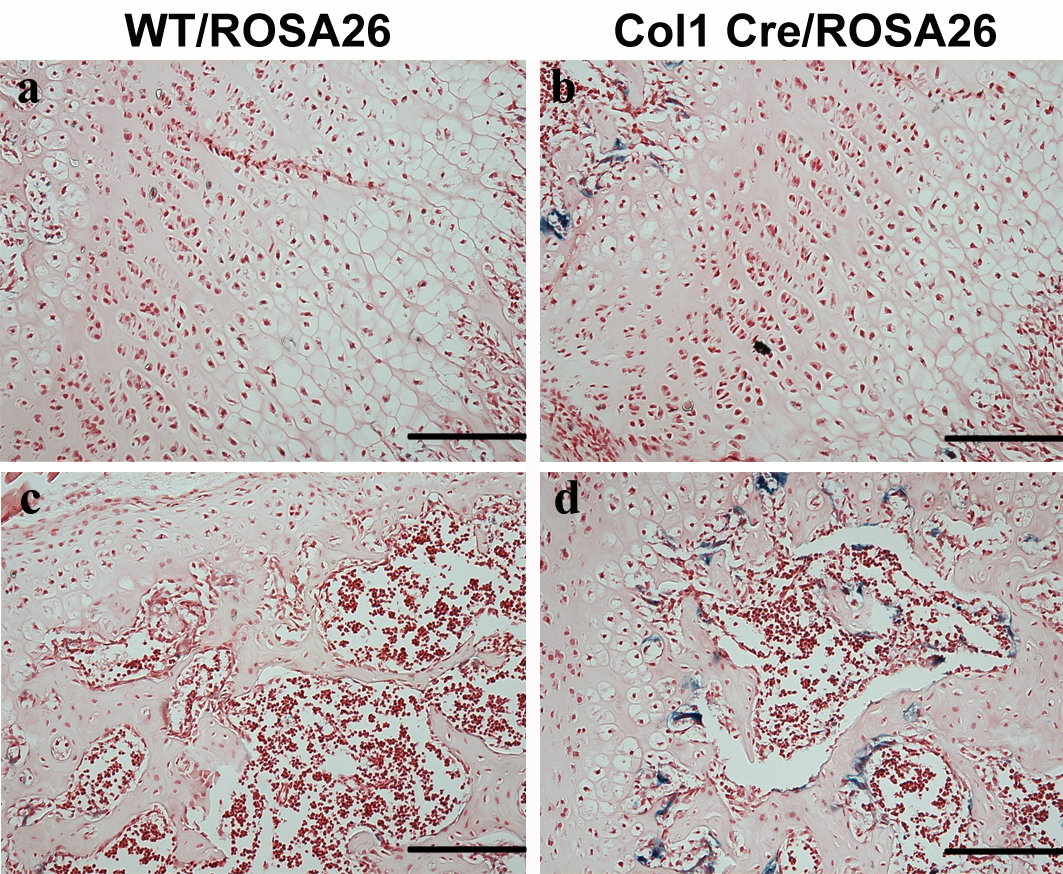

Supplement: Figure S1 — Site-specificity of Col1-CreERTM. a Col 1 Cre expression was not found in growth plate in wild-type mice crossed with ROSA26 mice. b Col 1 Cre expression was not found in growth plate in Col1-CreERTM mice crossed with ROSA26 mice. c Col 1 Cre expression was not found in osteoblasts in wild-type mice crossed with ROSA26 mice. d Col 1 Cre expression was found in osteoblasts in Col1-CreERTM mice crossed with ROSA26 mice. Bars: 200 µm. (TIF) [file pone.0074093.s001.tif]

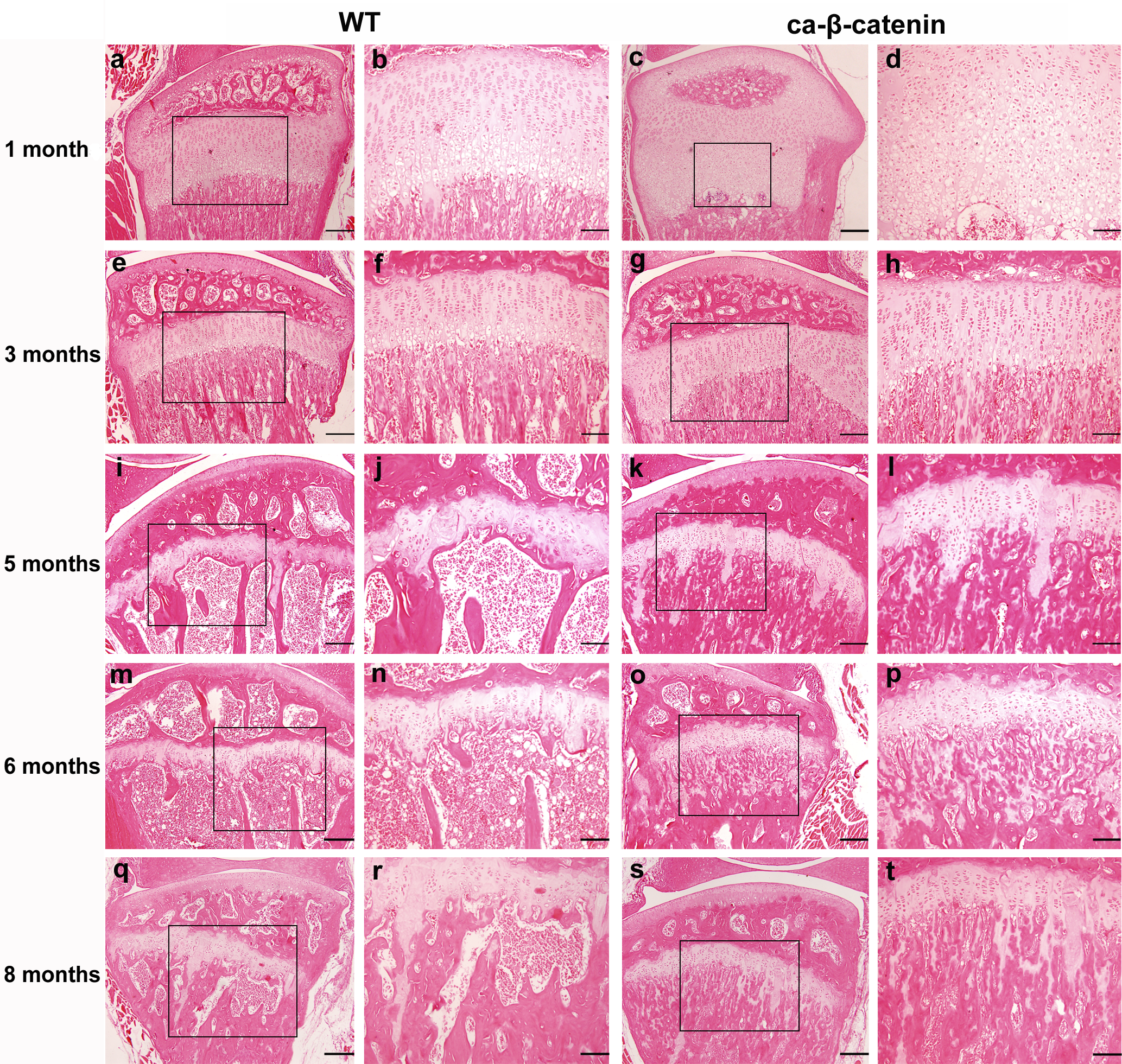

Supplement: Figure S2 — H&E staining of coronal sections of the tibia in wild-type and CA-β-catenin mice. Boxes in a, c, e, g, I, k, m, o, q and s are magnified in b, d, f, h, j, l, n, p, r and t, respectively. Bars: 200 µm (a, c, e, g, I, k, m, o, q and s) and 100 µm (b, d, f, h, j, l, n, p, r and t). (TIF) [file pone.0074093.s002.tif]

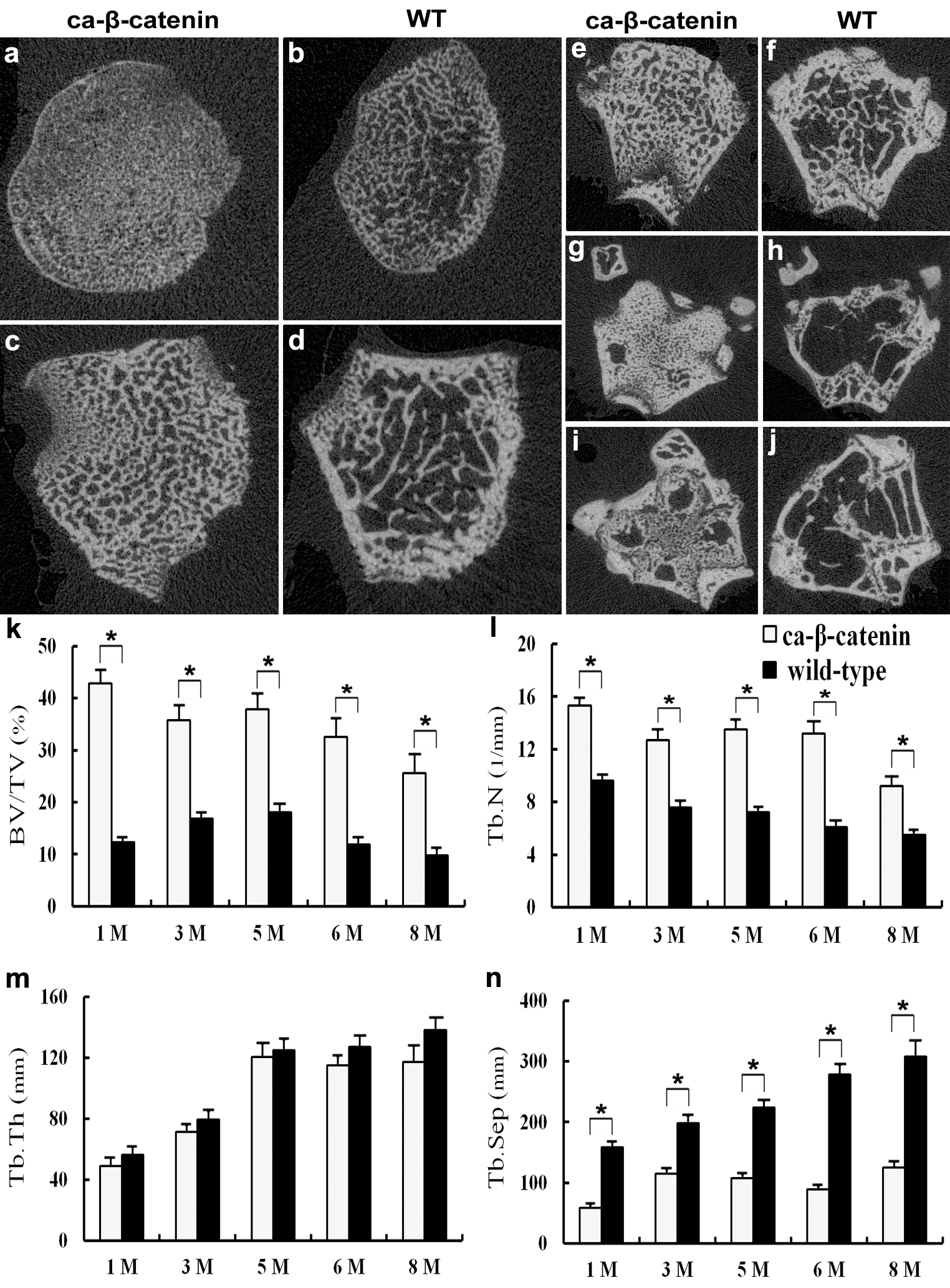

Supplement: Figure S3 — MicroCT examination of trabecular bone of proximal tibia in each group. a-j representative, transverse MicroCT images of the trabecular bone in CA-β-catenin and wild-type mice at 1 month (a and b), 3 months (c and d), 5 months (e and f), 6 months (g and h) and 8 months (i and j) of age. k MicroCT analysis of BVF (BV/TV, %) of the proximal tibia in CA-β-catenin mice and wild-type mice. BV trabecular bone volume (mm3); TV total volume selected for analysis (mm3). l Trabecular number (Tb.N) of the proximal tibia in CA-β-catenin and wild-type mice. m Mean trabecular thickness (Tb.Th) of the proximal tibia in CA-β-catenin and wild-type mice. n Trabecular separation (Tb. Sp) of the proximal tibia in CA-β-catenin and wild-type mice. Bars represent the mean ± SEM (n=6 for each group). * p<0.05. (TIF) [file pone.0074093.s003.tif]

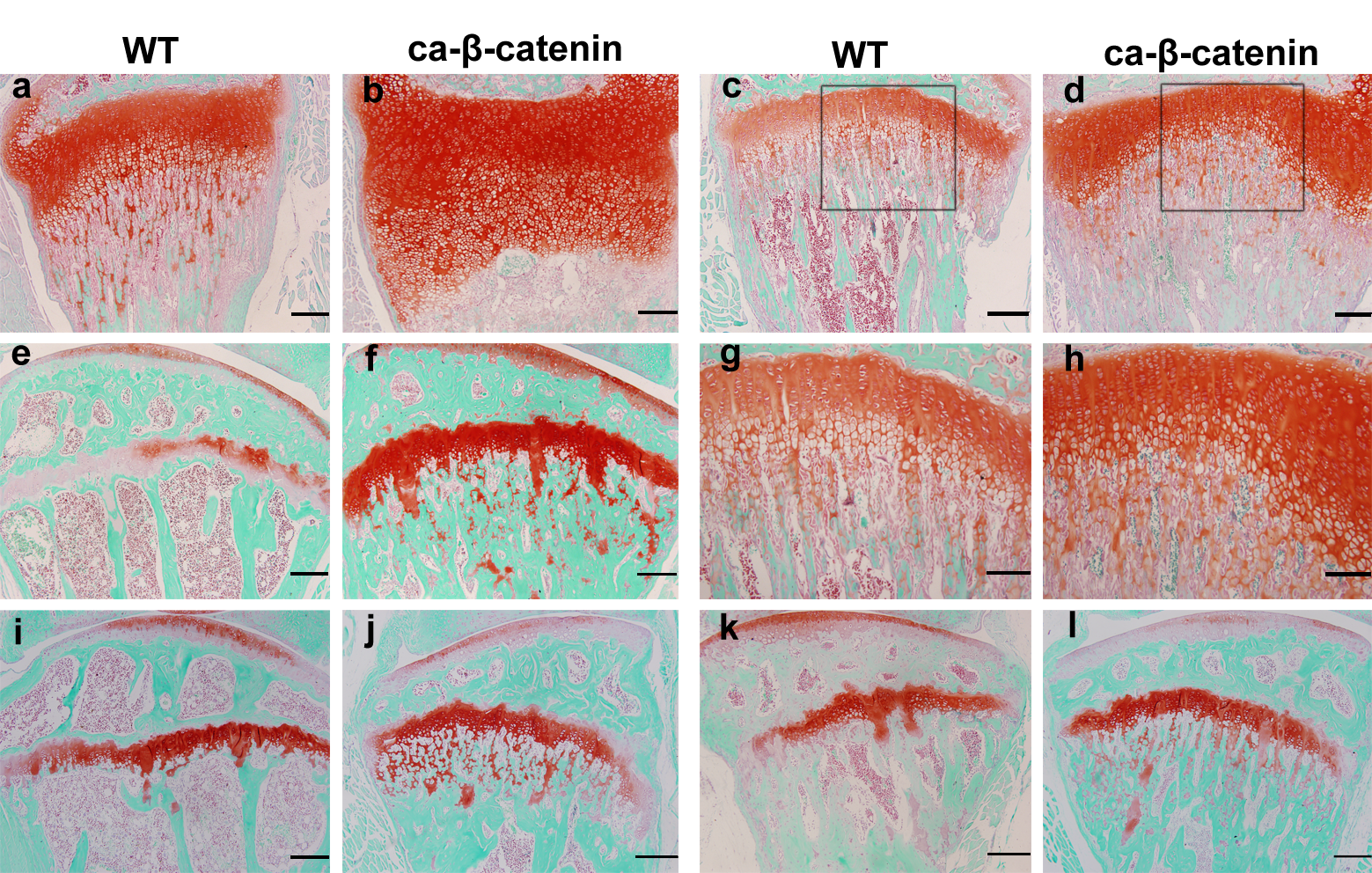

Supplement: Figure S4 — Safranin O staining of tibia in each group. a-h Safranin O staining of coronal sections of tibia in wild-type and CA-β-catenin mice at 1 month (a and b) 3 months (c,d g and h) 5 months (e and f), 6 months (i and j) and 8 months (k and l) of age. Boxes in c and d are magnified in g and h, respectively. Bars: 200 µm (a, b, c, d, e, f, i, j, k and l) and 100 µm (g and h). (TIF) [file pone.0074093.s004.tif]

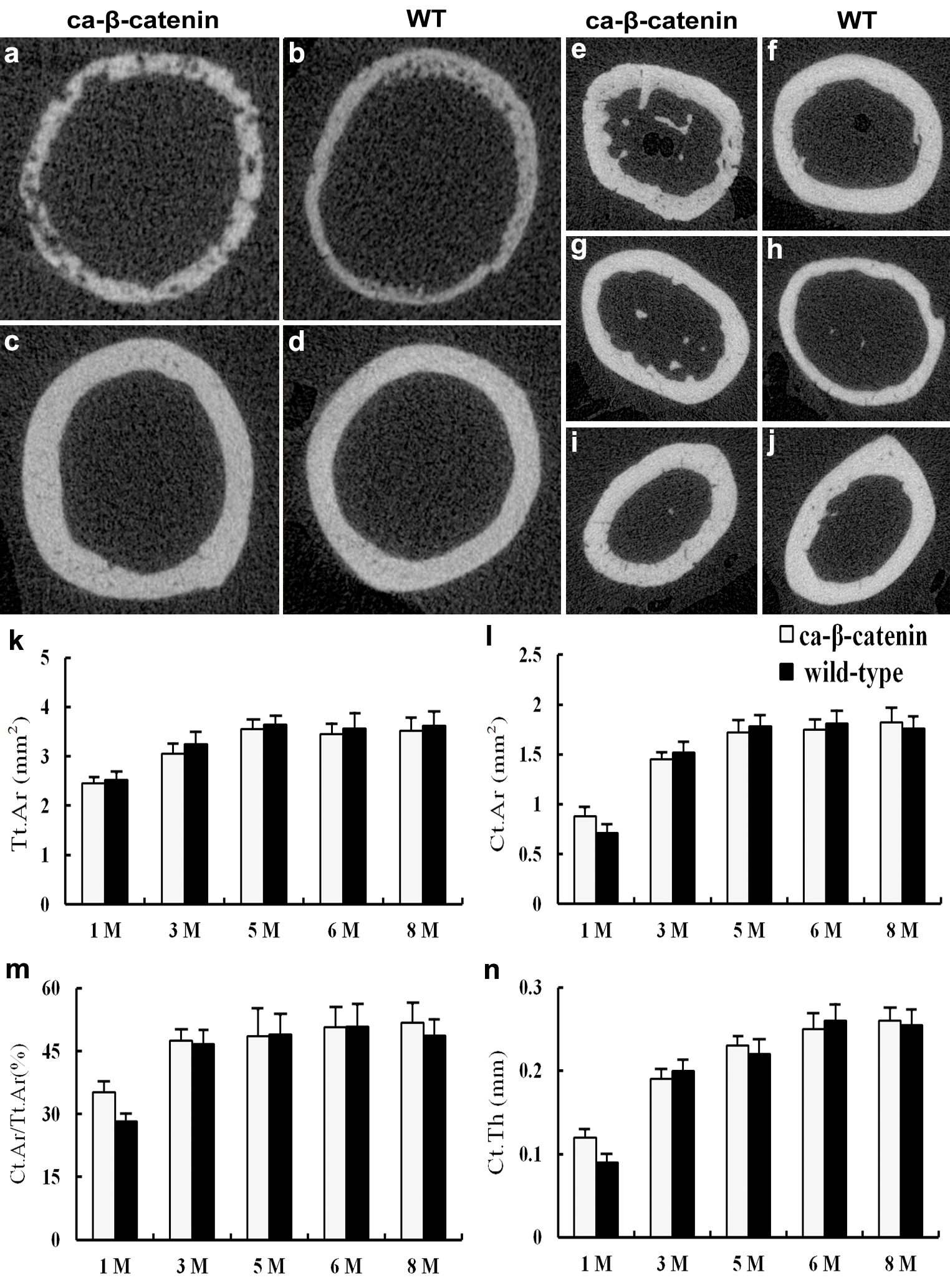

Supplement: Figure S5 — MicroCT examination of cortical bone of tibia in each group. a-j representative, transverse MicroCT images of the cortical bone in CA-β-catenin and wild-type mice at 1 month (a and b), 3 months (c and d), 5 months (e and f), 6 months (g and h) and 8 months (i and j) of age. k Total cross-sectional area (Tt.Ar) in CA-β-catenin and wild-type mice. l Cortical bone area (Ct.Ar) in CA-β-catenin and wild-type mice. m Cortical area fraction (Ct.Ar/Tt.Ar) in CA-β-catenin and wild-type mice. n Average cortical thickness (Ct.Th) in CA-β-catenin and wild-type mice. Bars represent the mean ± SEM (n=6 for each group). *p<0.05. (TIF) [file pone.0074093.s005.tif]

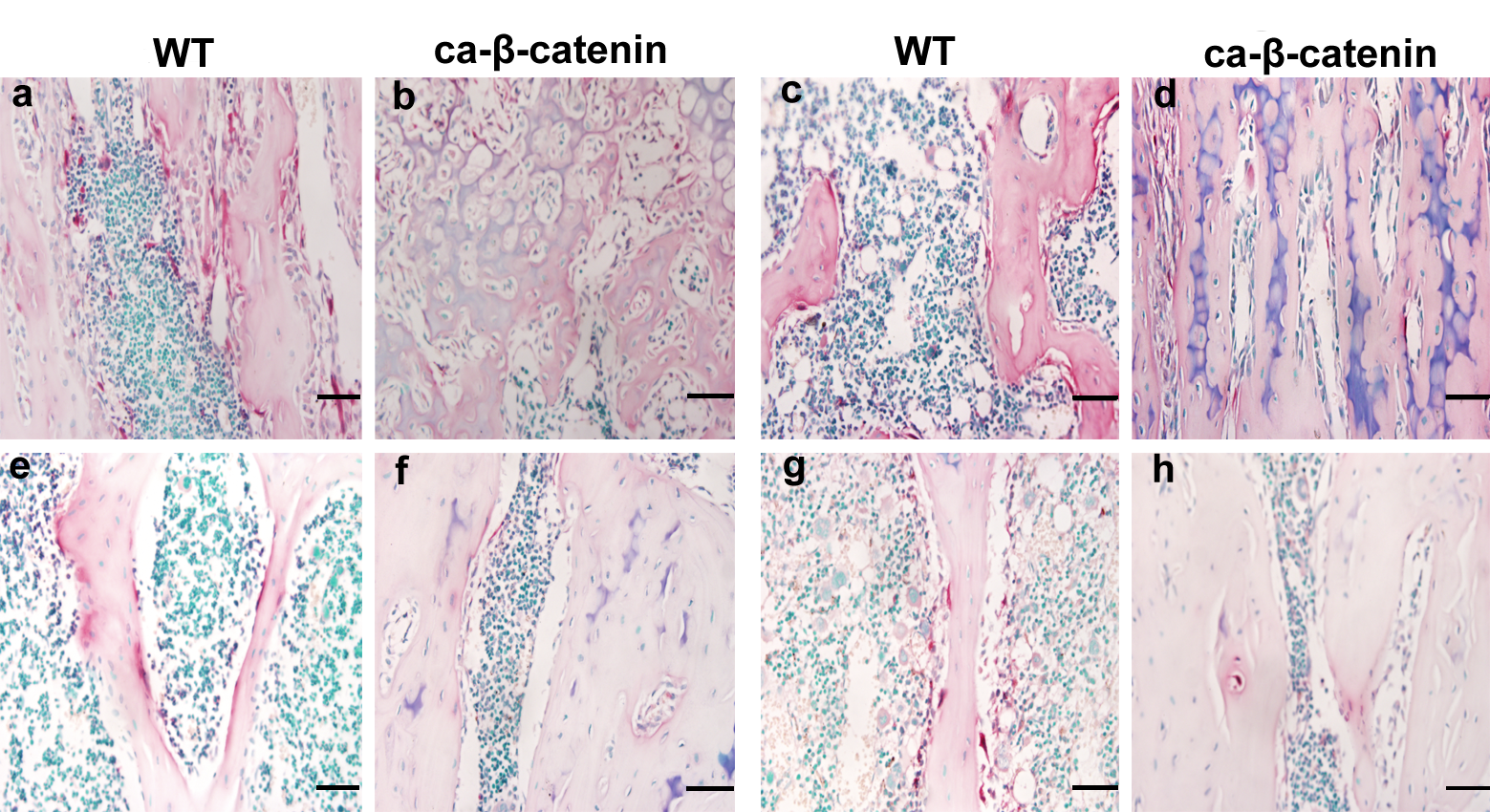

Supplement: Figure S6 — TRAP staining of tibia in each group. a-h TRAP staining of coronal sections of tibia in wild-type and CA-β-catenin mice at 1 month (a and b), 3 months (c and d), 6 months (e and f), and 8 months (g and h) of age. (TIF) [file pone.0074093.s006.tif]
